# The Policy of the Buffalo Medical Journal

**Published:** 1911-09

**Authors:** 


### The Policy of the Buffalo Medical Journal

THE change in the editorial management of the BUFFALO MEDICAL JOURNAL naturally raises the question among its readers, as to its future policy. Few periodicals of any kind have had so long an existence nor under such able management and, whatever the aspirations and the enthusiasm of a new incumbent may be, any attempt at radical change would indicate merely a failure to appreciate the worth of illustrious and lamented predecessors. A consistent and persistent effort will, however, be made to secure progressive improvement in details, increase in material contents and extension of territory.

It may be permissible to state that the acceptance of the responsibility of the JOURNAL by the present editor does not imply either the resignation of active practice nor the assumption of unfamiliar duties since he has had a quarter of a century's experience embracing nearly every detail of journalistic and editorial work of a medical nature.

The sole ultimate end of medical study is to prevent and cure disease. This end can be reached only by a thorough understanding both practical and scientific, of the nature of disease and of the means employed against it. The best type of physician is the one more interested in practising medicine than in running a medical society, formulating ethical principles or merely scientifically curious about the normal and abnormal phases of the animal, especially the human organism. Hence, the main function of a medical journal, unless of highly specialised nature, should be the discussion of practical problems in diagnosis, and methods of treatment, but with an intelligent basis of science which enables a broad grasp of principles. We ask, therefore, for original articles bearing as directly as possible on the actual work of the physician, avoiding superficiality on the one hand, abstruseness on the other. So far as is practicable, articles on related subjects will be grouped together, varying the topics discussed from issue to issue so as to cover the field of medicine and surgery generally. No fear need be entertained that the Journal will be unduly devoted to gastro-enterology in the future any more than it has been devoted to obstetrics and gynecology in the past.

The Buffalo Medical Journal owes its existence to the natural demand for an organ that shall be essentially local, though in no narrow sense either geographically or otherwise. This fact implies that the Journal should report the medical news of the region—not of Buffalo alone but of adjacent cities and the intervening country. Every reader should feel that any change in his residence, office hours, and the like, and every important event in his professional life are worth recording. We wish to announce in advance and to report in retrospect though necessarily briefly, the transactions of the various medical organisations of this region as well as of meetings and enterprises in which the profession is interested. Obituary notices also belong within this department and will have an increasing value historically as time passes. With no lack of respect for the dead, such notices must be brief, in consideration of the pressing needs of the living.

The editorial department should, except by reviews and compilations, avoid the presentation of problems of medical practice appropriate to the department of original communications, just as much as the latter should avoid the lengthy discussion of matters of ethics and policy. We do not believe that there is, at present, any issue confronting the profession as a whole, which involves a decision between right and wrong or whose determination, one way or the other, would seriously jeopardise the interests of the profession. As heretofore, the Buffalo Medical

JOURNAL will be independent in its editorial pages but as free as possible from personal views and interests. As to personal prejudice, in the narrow sense, we are glad to say that there is no person and no organisation within our scope for whom or which we have any but the kindest regard. It is perhaps, unnecessary to make this statement but we do so because the term *independence* is often used to imply a pile of stones ready to throw at every passer-by.

It is only fair that the readers of the JOURNAL should be equally free to express individual opinions on matters of professional policy and polity and, subject only to necessary limitations of space and the ordinary amenities of debate, our columns will always be open to correspondence on any topic of interest to the profession.

We ask the continuance of the cordial support which the profession has always accorded to the BUFFALO MEDICAL JOURNAL.

